# KCNMA1 cooperating with PTK2 is a novel tumor suppressor in gastric cancer and is associated with disease outcome

**DOI:** 10.1186/s12943-017-0613-z

**Published:** 2017-02-23

**Authors:** Gaoxiang Ma, Hanting Liu, Qiuhan Hua, Meilin Wang, Mulong Du, Yadi Lin, Yuqiu Ge, Weida Gong, Qinghong Zhao, Fulin Qiang, Guoquan Tao, Zhengdong Zhang, Haiyan Chu

**Affiliations:** 1 Department of Environmental Genomics, Jiangsu Key Laboratory of Cancer Biomarkers, Prevention and Treatment, Cancer Center, Nanjing Medical University, Nanjing, China; 2 Department of Genetic Toxicology, The Key Laboratory of Modern Toxicology of Ministry of Education, School of Public Health, Nanjing Medical University, Nanjing, China; 3 Department of General Surgery, Yixing Tumor Hospital, Yixing, China; 4 Department of General Surgery, The Second Affiliated Hospital of Nanjing Medical University, Nanjing, China; 5 Core Laboratory, Nantong Tumor Hospital, Nantong, China; 6 Department of General Surgery, Huai-An First People’s Hospital Affiliated to Nanjing Medical University, Huai-An, China; 7 Department of Environmental Genomics, School of Public Health, Nanjing Medical University, 101 Longmian Avenue, Jiangning District Nanjing, 211166 China

**Keywords:** Gastric cancer, KCNMA1 Methylation, Prognosis

## Abstract

**Background:**

Inactivation of tumor suppressor genes by promoter hypermethylation plays a key role in the tumorgenesis. It is necessary to uncover the detailed pattern of whole genome-wide abnormal DNA methylation during the development of gastric cancer (GC).

**Method:**

We performed a genome-wide methylation detection using 12 paired of GC tissues and their corresponding normal tissues. Methylation-specific PCR (MSP) and bisulphite sequencing (BSP) were used to measure methylation status of specific CpG site. Based on the bioinformatic analysis, the cell phenotypes and mouse model experiments were constructed to detect effect of the target gene. Using the Kaplan–Meier survival curve, the clinical value of *KCNMA1* was assessed in GC patients.

**Results:**

The CpG site cg24113782 located at the promoter of *KCNMA1* showed the most significant difference, contributing to the commonly silenced *KCNMA1*in gastric cancer cells and primary GC tissues. The promoter methylation of *KCNMA1* was detected in 68.7% (77/112) of tumor tissues, compared with 16.2% (18/112) of normal tissues (*P* < 0.001). The survival curve indicated that *KCNMA1* hypermethylation was significantly associated with the shortened survival in GC patients (*P* = 0.036). *KCNMA1* significantly inhibited biological malignant behavior of gastric cancer cell by inducing cell apoptosis in vitro, and suppressed xenograft tumor growth in subcutaneous mouse models (both *P* < 0.001). Furthermore, the anti-tumor effect of *KCNMA1*was mediated through suppressing the expression of *PTK2*.

**Conclusion:**

*KCNMA1* is a critical tumor suppressor in gastric carcinogenesis and its hypermethylation is an independent prognostic factor in patients with gastric cancer.

**Electronic supplementary material:**

The online version of this article (doi:10.1186/s12943-017-0613-z) contains supplementary material, which is available to authorized users.

## Background

Gastric cancer (GC) is one of the most common malignancies and remains the second leading cause of cancer-related death worldwide. Despite modified surgical and adjuvant treatment strategy, the prognosis of GC patients is poor, with a 5-year overall survival of less than 25% [1, 2]. There are considerable evidences indicating that epigenetic alterations, particularly inactivation of tumor suppressor genes through promoter hypermethylation, play an important role in the development and progression of GC [3]. Identification of such novel genes targeted by promoter hypermethylation may provide insights into alternative approaches for diagnostic and therapeutic targets and the epigenetic mechanisms in GC. In normal cells, the pattern of DNA methylation is handed down to the daughter cells during mature cell division. However, the aberrant alterations in the DNA methylation profile of mature cells are frequently observed in many human cancers, including GC [4, 5]. Therefore, identification of the differences of the DNA methylation status in GC to reveal the role of epigenetic instability on the initiation and progression of GC is necessary.

To uncover the genome-wide DNA methylation profiles of GC in a more comprehensive way, we performed a microarray analysis between gastric cancer issues and their matched normal tissues with Illumina Infinium Human Methylation450 BeadChip array that include >485,000 CpG sites distributed throughout the genome [6]. We found that the gene, potassium channel, calcium activated large conductance subfamily M alpha, member 1(*KCNMA1*), the function of which remains largely unexplored, was moderated by promoter methylation in gastric cancer. *KCNMA1* (also named BK) potassium channels are a diverse class of ion channels expressed in many different cell types [7]. The protein encoded by *KCNMA1* represents the voltage and Ca^2+^-activated K^+^ channel, and is involved in the feedback inhibition of the action potential frequency and Ca^2+^ influx [8, 9]. Emerging evidences have identified that the Ca^2+^ is closely related to cell apoptosis [10, 11]. Moreover, by bioinformatics analysis based on The Cancer Genome Atlas (TCGA), we found the *KCNMA1*could regulate the expression of *FAK* (focal adhesion kinase), also named *PTK2*, which is a non-receptor tyrosine kinase and moderate cancer proliferation, migration and survival [12]. And it may regulate the cell apoptosis by PI3K-AKT pathway [13]. It is possible that the Ca^2+^ is involved in apoptosis by cooperating with *PTK2*.

We reasoned the *KCNMA1* contribute to the GC risk by regulating the key apoptosis gene *PTK2*. In this study, therefore, we set out to explore the expression profile, epigenetic regulation, biological function, molecular basis and clinical application of *KCNMA1* in GC.

## Methods

### GC cell lines

A total of four GC cell lines (i.e., MGC-803, BGC-823, SGC-7901, and MKN-28) and one normal human gastric epithelial cell (GES-1) were used in this study. All cell lines were maintained in RPMI-1640 medium (Gibco BRL, Rockville, Maryland, USA) with 10% fetal bovine serum (Gibco BRL). And the identity of the cell lines were confirmed by short tandem repeat (STR).

### Gastric tissue samples

Seventy-nine paired tumor and adjacent non-tumor gastric samples were obtained from GC patients at the Second Affiliated Hospital of Nanjing medical University in Nanjing, China. A total of 75 patients with histologically-confirmed gastric cancer and adjacent non-tumor tissues were evaluated for *KCNMA1* with real-time PCR (RT-PCR) and 112 patients with methylation-specific PCR (MSP). The 75 paired of GC tissues were mainly collected from The Second Affiliated Hospital of Nanjing Medical University, and 112 GC tissues were from the First Affiliated Hospital of Nanjing Medical University without paired adjacent tissues. All subjects of this study signed informed consent for obtaining the study specimens.

### Genome-wide Methylation Profiling

DNA methylation analysis was performed by Shanghai Genergy Co. Ltd (Shanghai, China) using the Illumina Human Methylation450 BeadChip (Illumina). These arrays contain probes for approximately 450,000 CpG loci sites. Target was prepared and hybridized according to the “Illumina Infinium HD Methylation Assay, Manual Protocol”. The methylation level was computed as a β value according to the normalized probe fluorescence intensity ratios between methylated and unmethylated signals: β value = signal intensity of the methylated allele (sum of signal intensity of the unmethylated and methylated allele + 100). The DNA methylation level for each interrogated CpG site was evaluated as a β value, which ranged from 0 (not methylated) to 1 (fully methylated). The significant *P* values of the normal tissue and tumor tissue groups were calculated by paired Wilcox non parametric test, and the Benjamini and Hochberg method were used to carry out multiple test correction calculation FDR [14]. We chose the maximum difference of β value between the normal tissue and tumor tissue groups in further research.

### RNA extraction and Quantitative real-time PCR (qRT PCR)

The total RNA was extracted from tissues using Trizol reagent (Invitrogen, CA, USA). The cDNA was synthesized using M-MLV reverse transcriptase (Invitrogen) after RNA extraction according to the manufacturer’s instruction. The expression level of genes was detected by qRT-PCR using SYBR Green assays (TaKaRa Biotechnology, Dalian, China). Glyceraldehyde 3-phosphate dehydrogenase (*GAPDH*) was chose to act as an internal control, and the assay was conducted by ABI 7900 system (Applied Biosystems, CA, USA). To evaluate the primer efficiency, we have used the standard curve to calculate the amplification efficiency. The amplification efficiency of GAPDH, KCNMA1 and PTK2 was 98.1, 96.3 and 97.5% respectively. The expression of each gene was quantified according to fold change using 2^−ΔΔCt^ methods. The primers sequences are available in Additional file 1: Table S1.

### DNA extraction, MSP and BSP

The DNA of tissues was obtained using E.Z.N.A ™ tissue DNA kit (Omega Bio-Tek. USA). Then the tissue DNA was modified by EZ DNA Methylation-Gold™ Kit (Zymo Research) according to the manufacturer’s instruction. The MSP and BSP primer was designed by the Methyl Primer Express v1.0 (Applied Biosystems), as shown in Additional file 1: Table S1.

### Construction of *KCNMA1* expression plasmid and RNA interference

The full-length open reading frame sequence of *KCNMA1* was constructed by GenScript USA Inc. (Nanjing, China) and then was subcloned into the mammalian expression vector pIRES-EGFP. The product was verified by DNA sequencing. Three small interfering RNA (siRNA) were synthesized to target *PTK2* (RiboBio, Guangzhou, China). After detection of the interference efficiency, si-PTK2-2 (named si-PTK2 in this study) had the optimal efficiency and was selected for the following study. The sequences are shown in Additional file 1: Table S1. GC cells, MGC-803 and BGC-823, were transiently transfected with the *KCNMA1* over-expression plasmid and si-PTK2 using Lipofectamine 2000 (Invitrogen, Carlsbad, CA, USA) transfection reagent according to the instruction. The pIRES-EGFP empty vector was used as negative control (NC).

### The malignant behaviors of cancer cells

Using GC cells, we performed a series of assays to detect the effects of *KCNMA1* on the malignant behaviors including apoptosis assay, proliferation, colony formation and migration. The detail of assay conditions was shown in Additional file 2.

### Subcutaneous xenograft models in vivo

MGC803 cells (1 × 10^7^cells in 0.2 ml PBS) that was stably transfected with *KCNMA1* expression vector or empty vector were subcutaneously injected into the dorsal right flank of 5-week-old male Balb/c nude mice (*n* = 10 per group). The tumor diameter in the nude mice was measured every 2 days for 2–3 weeks. After 20 days, all mice were sacrificed and the tumor weight and size were measured. The experiment was approved by the Animal Ethics Committee of Nanjing Medical University.

### Statistical analysis

The independent or paired *t* test was used to calculate the difference between two preselected groups or paired samples. The associations between the *KCNMA1* methylation and expression and clinic opathological characteristics of GC patients were compared using Pearson’s *χ* ^2^ test. The Kaplan Meier survival curves and log-rank test were used to evaluate the relation between the overall survival and methylation status. The *P* < 0.05 was regarded as statistical significance.

## Results

### Identification of methylation status between gastric cancer tissues and normal tissues

**Fig. 1:**
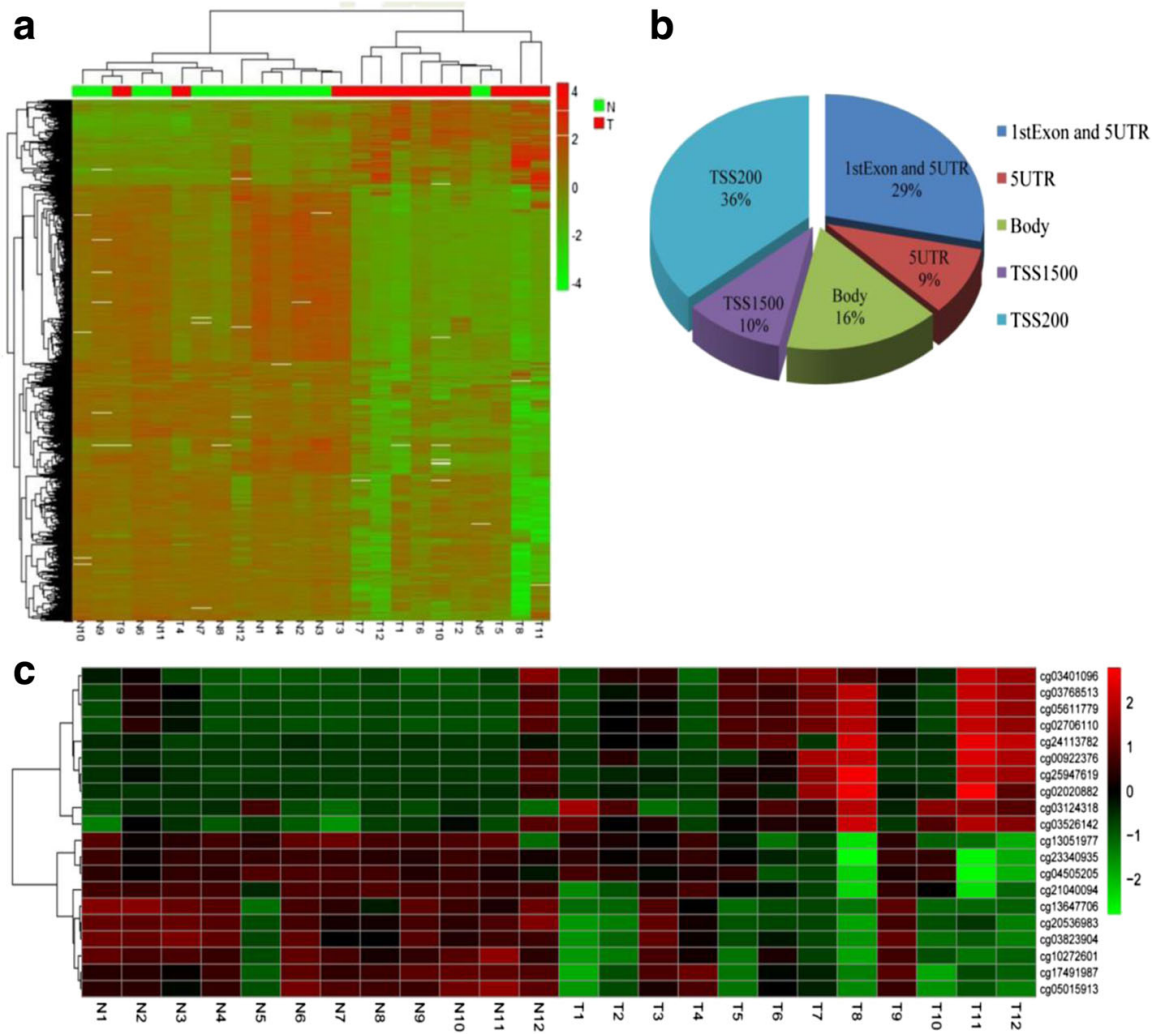
Hierarchical clustering analysis of the microarray assay. **a** The heat map of the different methylated site between the gastric carcinoma and paired corresponding normal tissues. **b** The gene location of the most 100 significantly hypermethylated CpG sites. **c** Hierarchical clustering analysis on the most 10 significantly hypermethylated and hypomethylationCpG sites. N, normal tissues, T, gastric carcinoma tissues. TSS1500, 1500 bases before the transcription start site. TSS200, 200 bases before the transcription start site. Body, the intron and exon of gene

Twelve paired of the tumor and the paired normal tissues were profiled (Additional file 1: Table S1). Results of hierarchical clustering analysis on the most significantly hypermethylation CpG site are shown in Fig. 1a. This analysis revealed a remarkable segregation between the tumor and the paired normal tissues. Through further analysis, we found that the most of top 100 hypermethylation site locate the promoter of the genes (Fig. 1b). And the top 10 high methylated CpG sites can well distinguish the tumor tissues from the normal tissues (Fig. 1c). Interesting, the CpG site cg24113782 with most significant difference was located in the promoter region of *KCNMA1*. Moreover, this result was also supported by the data from the independent TCGA data. The above results indicated cg24113782 had a notably high β-score value in the cancer tissues compared normal tissues (*P* < 0.001) (Additional file 3: Figure S1). In addition, this finding was also identified in the Human Methylation 27 array from TCGA, which has a low density and mainly focuses on CpG-sites mapping to gene promoter regions. Although the cg24113782 site was not included in the HumanMethylation 27 array, we found the other CpG site cg04688368 in the HumanMethylation27 array which also located on the *KCNMA1* promoter region. The β-score value of cg04688368 between tumor and paired normal tissues had a significant difference in the paired GC tissue (*P* < 0.001, Additional file 3: Figure S1).

### Silence or downregulation of *KCNMA1* by promoter methylation in gastric cancer cells and tissues

The expression of *KCNMA1* was detected in the GC cells (i.e., MGC-803, BGC-823, MKN-82, SGC-7901) and the normal human gastric epithelial cell line (GES1) using RT-PCR (Additional file 3: Figure S2). The mRNA expression of *KCNMA1* was silenced or reduced in the GC cells compared with normal human gastric epithelial cell. To identify whether the cancer cell methylation directly mediates *KCNMA1* expression, we treated the two cell lines (i.e., MGC-803 and BGC-823) with the demethylation agent, 5-Aza-2′-deoxycytidine (5-Aza; Sigma-Aldrich), for 72 h. Notably, this treatment restored expression of *KCNMA1* in the two silenced cell lines (Additional file 3: Figure S2), suggesting that the expression silence of *KCNMA1* was moderated by the aberrant promoter methylation.

**Fig. 2:**
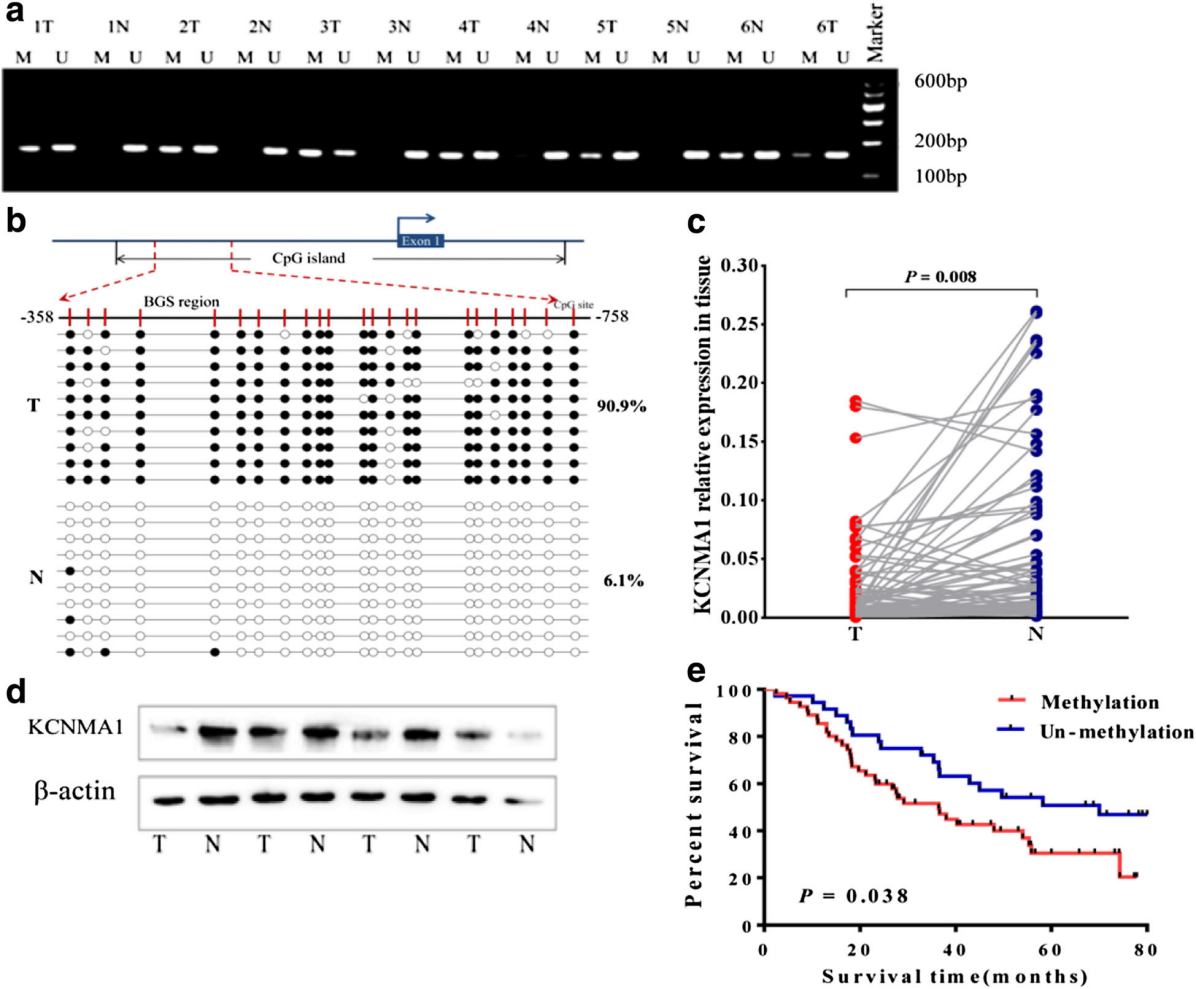
The difference of KCNMA1 methylation and expression between the gastric carcinoma and paired corresponding non-cancerous tissues, and the prognosis value. **a** Analysis of promoter methylation of KCNMA1 in gastric tumor tissues and paired corresponding normal tissues. The presence of PCR products in lane M indicates the presence of methylated alleles, and in lane U indicates the presence of unmethylated alleles. *N* = non-tumor, T = tumor; **b** BGS analysis also confirmed high levels of promoter methylation in a paired carcinoma tissues and corresponding normal tissues; Level of KCNMA1 mRNA (**c**) and protein (**d**) in the carcinoma and adjacent tissue; **e** The influence of KCNMA1 methylation on GC patients prognosis. N, normal tissues, T, gastric carcinoma

To detect the contribution of promoter methylation to the down-regulation of *KCNMA1* for tumor and paired normal tissues, methylation status of its promoter was examined by methylation-specific PCR (MSP) in 112 paired tissues. We found 68.7% (77/112) GC tissues were methylated, but only 16.2% (18/112) normal tissues were methylated (Fig. 2a), and the BSP results also confirmed this finding (Fig. 2b). In addition, we detected the expression of *KCNMA1* in 75 paired of cancer and normal tissues. The expression level of *KCNMA1* in cancer tissues was significantly decreased compared with normal tissues (*P* = 0.008, Fig. 2c, d). The same result was found in TCGA and GEO data (Additional file 3: Figure S1). As shown in Table 1, the aberrant *KCNMA1* methylation status in GC tissues was associated with tumor sizes and depth of invasion. Meanwhile, we found the aberrant expression contributed to the tumor sizes in Table 2.

**Table 1:** Clinicopathological features of KCNMA1 promoter methylation in 112 patients with GC

Factors	Methylated (*N* = 77)	Non-methylated (*N* = 35)	*P* value
Age (mean ± SD)	64.44 ± 1.00	61.81 ± 1.86	0.182
Gender			
Male	50	22	0.879
Female	27	13	
Tumor sizes			
≤ 5 cm	35	26	**0.005**
> 5 cm	42	9	
Depth of invasion			
T1+ T2	17	2	**0.032**
T3+ T4	60	33	
Lymphnode metastasis			
N0	22	7	0.808
N1	23	12	
N2	17	9	
N3	15	7	
Metastasis			
M0	67	29	0.560
M1	10	6	
TNM stages			
I	9	2	0.687
II	19	11	
III	38	16	
IV	11	6	

The entries in bold showed the *P* value is less than 0.05

**Table 2:** The relationship between KCNMA1 expression and clinicopathological feature of 75 GC patients

Clinicopatholocical variables	Number of each group	KCNMA1 expression		*P* value
		High	Low	
Age(years)				
< 60	27	13	14	0.878
≥ 60	48	24	24	
Sex				
Male	62	30	32	0.720
Female	13	7	6	
Tumor size				
≤ 5 cm	42	26	16	**0.014**
> 5 cm	33	11	22	
Tumor site				
Cardia	26	12	14	0.922
Non-cardia	47	24	23	
Histological type				
Diffuse	41	21	20	0.721
Intestinal	34	16	18	
Depth of invasion				
T1 + T2	15	4	11	0.179
T3 + T4	60	20	35	
Lymph nodedistant metastasis				
N0 + N1	20	11	9	0.554
N2 + N3	55	26	29	
Distant metastasis				
M0	62	30	32	0.720
M1	13	7	6	
TNM				
I + II	22	10	12	0.665
III + IV	53	27	26	

The entries in bold showed the *P* value is less than 0.05

### *KCNMA1* is an independent predictor of prognosis in patients with GC

The association between *KCNMA1* methylation status and clinical outcome was analyzed in 91 patients with GC with known survival data. As shown in Fig. 2e, GC patients with *KCNMA1* methylation had significantly shorter survival than others (*P* = 0.038, log-rank test).

### Ectopic expression of *KCNMA1* suppressed GC cell proliferation, migration, invasion and colony formation

**Fig. 3:**
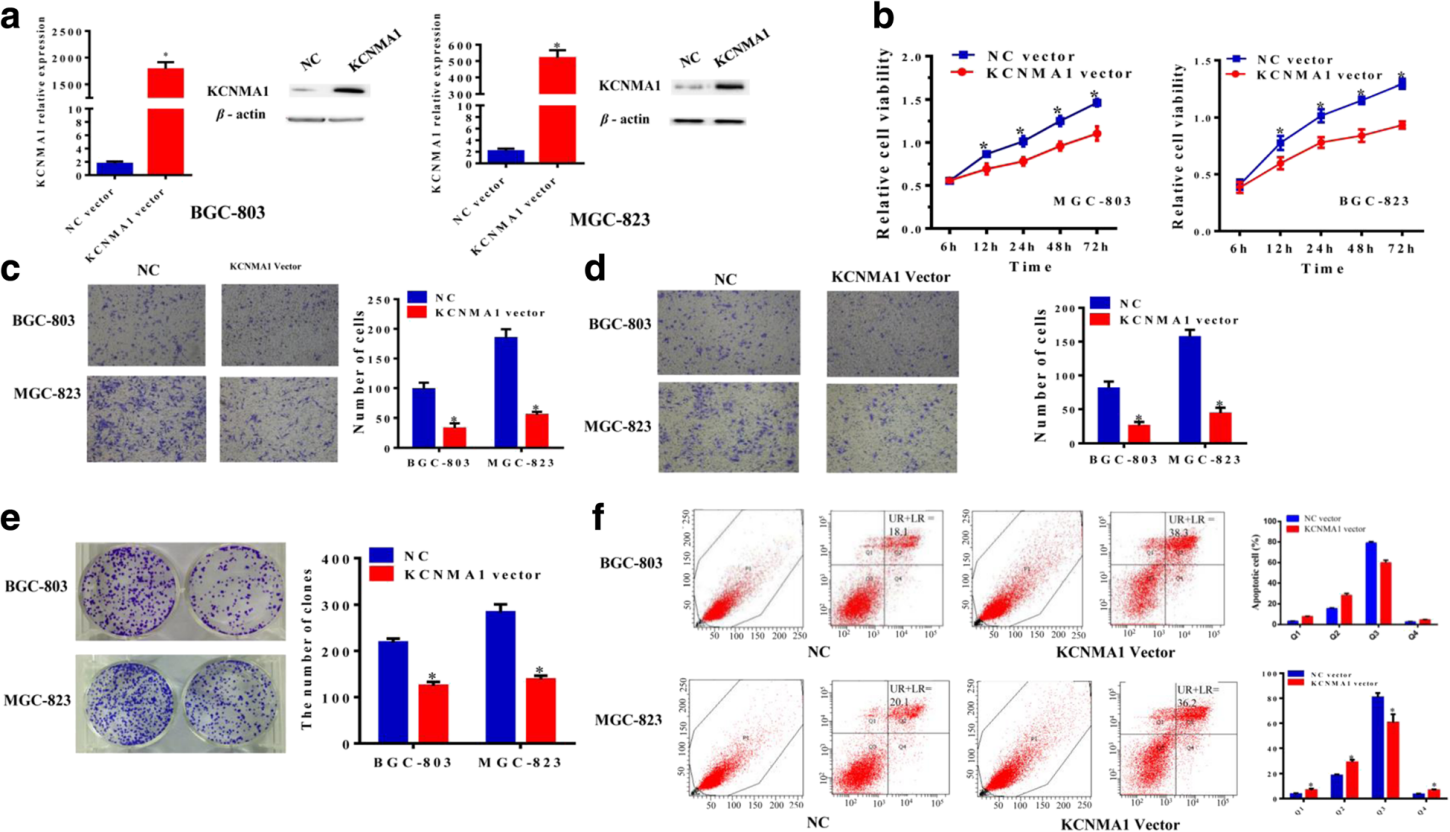
In vitro gain-function assays on KCNMA1. **a** Ectopic expression of KCNMA1 in BGC803 and MGC823 cells at mRNA and protein levels was confirmed by RT-PCR and western blot analysis. **b** KCNMA1 significantly inhibited cell viability. **c** Representative images of invasion assays for BGC803 and MGC823 cells transfected with control and KCNMA1 vector, error bars, s.d. *n* = 3 technical replicates. **d** Representative images of a migration assay for BGC803 and MGC823 cells transfected with control and KCNMA1 vector, error bars, s.d. *n* = 3 technical replicates. **e** KCNMA1 significantly inhibited cell colony formation ability. **f** KCNMA1 induces the apoptosis of BGC803 and MGC-823. (*F_left*) The cells were cultured for 48 h, the level of apoptosis was determined by flow cytometry, representative data from one of the three experiments was shown. (*F_right*) After MGC-823 transfected with KCNMA1, UR percentage + LR percentage in NC are less than cell treat with KCNMA1.*P < 0.01

Considering frequent silencing of *KCNMA1* in primary cancers and GC cell lines but not in normal gastric tissues, it suggested that *KCNMA1* may act as probably a tumor suppressor. *KCNMA1*-expressing plasmid was stably transfected into MGC803 and BGC823 cells. Re-expression of *KCNMA1* was confirmed by RT-PCR and Western blot analysis (Fig. 3a). Firstly, CCK-8 assay showed that proliferation of MGC803 and BGC823 cells were remarkably suppressed after *KCNMA1*over-expression for 24 h, 48 h and 72 h compared with those transfected with NC vectors (Fig. 3b). Compared with MGC803 and BGC823 cells transfected with NC vector, the cells with over-expression of *KCNMA1* for 48 h showed significantly decreased migration ability (*P* < 0.01, Fig. 3c). Besides, the suppression effect on invasion was also observed in both the two cells after 48 h of transfection (*P* < 0.01, Fig. 3d). Moreover, the inhibitory effect on GC cell growth was further confirmed by colony formation assay. The colonies formed by *KCNMA1*-transfected cells were significantly smaller and fewer than those formed by NC vector-transfected cells (*P* < 0.01, Fig. 3e).

### KCNMA1 induced cell apoptosis

**Fig. 4:**
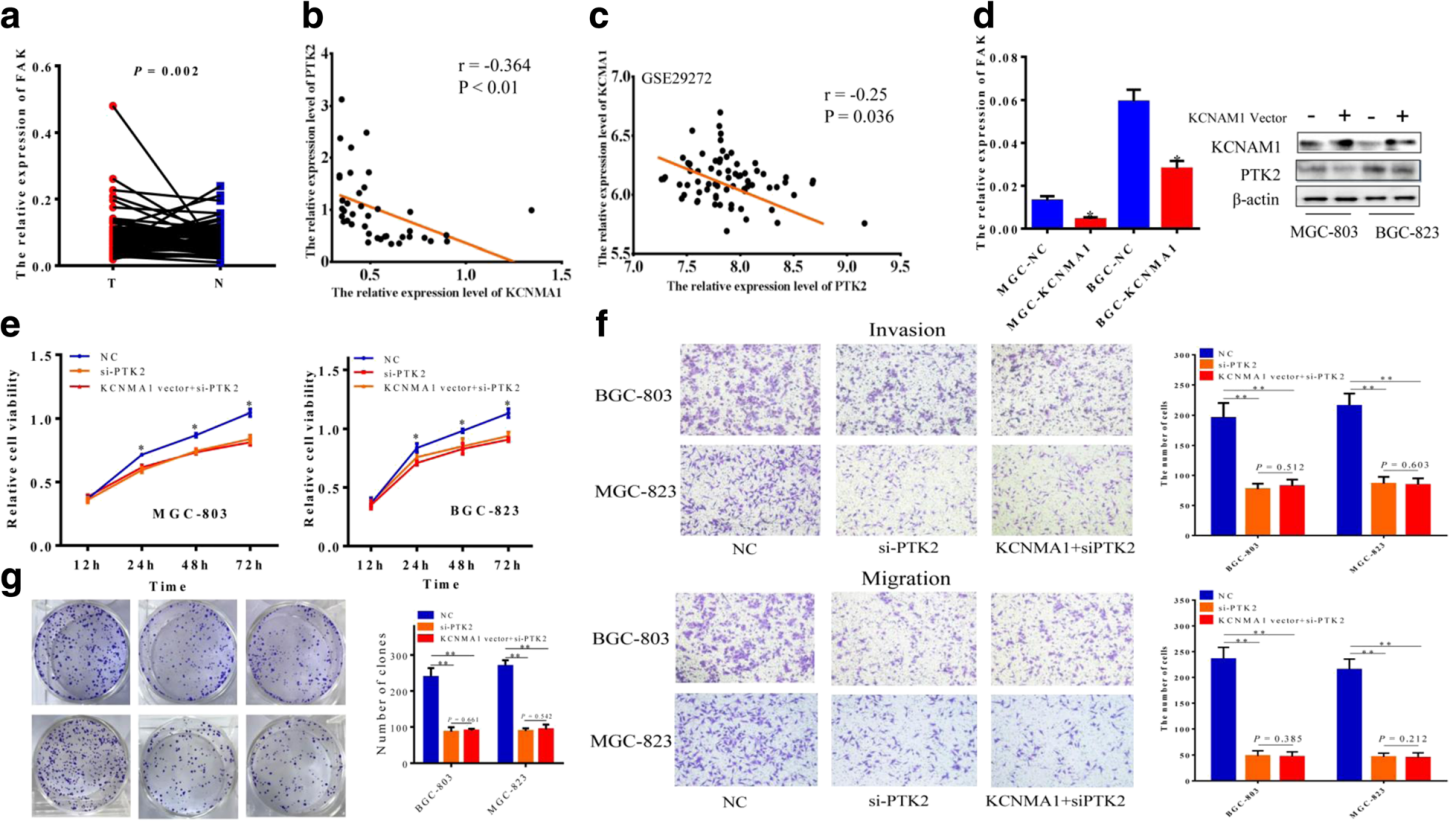
The association between KCNMA1 and PTK2, and Repeating observation on malignant cell behavior after co-transfected with NC, KCNMA1 vector and si-PTK2. **a** The expression of PTK2 in gastric carcinoma and paired corresponding normal tissues. **b** Expression levels of KCNMA1 and PTK2 in tissues were significantly correlated in a negative direction. **c** The correlation of KCNMA1 and PTK2 was verified in GEO (GSE29272). **d** The KCNMA1 suppress the expression of PTK2, and the relation were identified in BGC803 and MGC823 cells at mRNA and protein levels was confirmed by RT-PCR and western blot analysis. Inhibitory role of KCNMA1 on malignant cell phenotype, i.e. **e** invasion and migration; **f** proliferation and (**g**) colony formation; was diminished after PTK2 was knockdown.*P < 0.01, ** *P* < 0.001

Suppression of tumor cell growth is usually involved in concomitant activation of cell apoptosis pathways. Therefore we detected the contribution of apoptosis to the growth inhibition of *KCNMA1*over-expression cells using flow cytometry (Fig. 4f). The results indicated an increase in the numbers of both early apoptotic cells (*P* < 0.01) and late apoptotic cells (*P* < 0.01) in *KCNMA1*-transfected MGC803 and BGC823 cells compared with those transfected with NC vector (Fig. 4f).

### Identification of genes modulated by *KCNMA1* in GC cell lines

To gain insights into the molecular basis of apoptosis *KCNMA1*-modulated, the downstream target genes were characterized through cBioPortal for Cancer Genomics (Additional file 3: Figure S3) and found that the *PTK2* gene involved in FAK apoptosis pathways may be correlated with *KCNMA1*. Firstly, we found that the *PTK2* was significantly high expression in tumor tissues than paired normal tissues (Fig. 4a). Then, the correlation between the *KCNMA1* and *PTK2* was examined in gastric cancer tissues, and the result indicated that the expression levels of *KCNMA1* and *PTK2* were significantly correlated in a negative direction (*r* = −0.364, *P* < 0.01, Fig. 4b), which was further confirmed by the GEO data (*r* = −0.25, *P* = 0.036, Fig. 4b) (GSE29272). Taking into consideration the published researches, the function of antitumor of *PTK2* was also found in this study (Fig. 4e, f, g and Additional file 3: Figure S5). When KCNMA1-expressing plasmid was transfected into MGC803 and BGC823 cells, the expression level of *PTK2* was detected by RT-PCR and western blotting. As shown in Fig. 4d, the expression of *PTK2* had a significant decrease compared with NC cells.

### Knockdown of *PTK2* expression by siRNA

In order to identify whether the observed antitumor effects of *KCNMA1* was the consequence of its down-regulation of *PTK2* gene, knockdown of *PTK2* expression was achieved by siRNA interference. RT-PCR results of the interfered cells indicated that *PTK2* expression was remarkable decrease, except for si-PTK2-1 (Additional file 3: Figure S4). In the further study, si-PTK2 with the highest inhibition ratio up to 75% was selected. Malignant phenotypes of MGC803 and BGC823 cells were monitored repeatedly with both *KCNMA1* over-expressing and *PTK2* knockdown.

### Repeating observation on cell phenotype after KCNMA1-expressing plasmid and si-PTK2 transfected

In the repeated CCK-8 assay, we found that suppressed role of *KCNMA1*on GC cells proliferation was markedly weakened with co-transfection of *KCNMA1* vector and si-PTK2. As the presented in Fig. 4e, there was no significant difference in proliferation ratio of treated BGC823 and MGC803cells in the co-transfection of *KCNMA1* vector and si-PTK2 groups, compared with only the si-PTK2 groups. Similarly, inhibitory ability on gastric cancer cell migration and invasion was also attenuated by si-PTK2, that is, *KCNMA1* did not have the ability to suppress migration and invasion of gastric cancer cells after *PTK2* was knockdown (Fig. 4f). Furthermore, the differences were not found on inhibiting the cell colony formation between *KCNMA1* vector and si-PTK2 co-transfected cells and cells with transfection of si-PTK2 groups (Fig. 4g).

### *KCNMA1* repressed the growth of subcutaneous xenograft tumours in nude mice

**Fig. 5:**
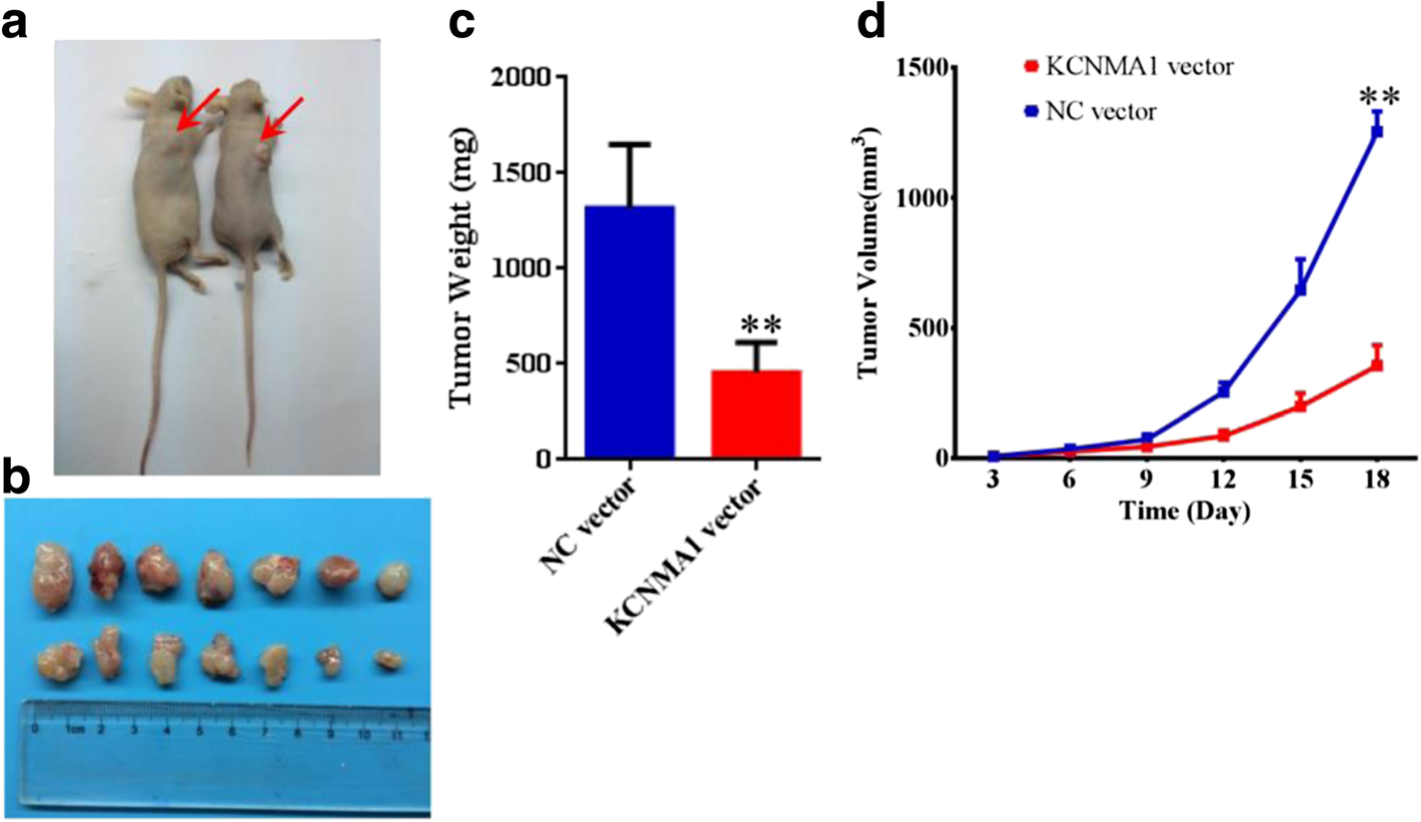
KCNMA1 suppresses gastric cancer cell growth in xenograft mice. **a** Representative burdened nude mice in KCNMA1 re-expressed and NC in MGC803 cells. Red arrows show position of subcutaneous tumors. **b** Representative xenografts in KCNMA1 re-expressed and NC in MGC803 cells. **c** Tumor weight in nude mice at the 18 day after inoculation of KCNMA1 NC and re-expressed MGC803 cells. Bars: mean of 7 mice. **d** The tumor volumes for KCNMA1 NC and re-expressed MGC803 cell xenografts. Points: mean of 7 mice. **P < 0.001

The subcutaneous xenograft tumor models were used to explore the effect of *KCNMA1* on gastric tumor cell growth in vivo. The empty vector transfected and subcutaneously injecting *KCNMA1*-transfected MGC803 cells were inoculated in nude mice. Then the status of subcutaneous tumor growth was recorded and monitored in the two groups. As shown in Fig. 5a, b and d, *KCNMA1*can significantly attenuates the growth of tumor volume and tumor volumes were compared with control cells (*P* < 0.001). And compared with NC cells at termination of the experiment, the weight of tumors with *KCNMA1*-transfected cells was also significantly reduced (*P* < 0.001, Fig. 5c).

## Discussion

In the present study, we have identified that *KCNMA1* is commonly silenced or down-regulated in primary gastric cancer tissues and gastric cancer cell lines due to promoter hypermethylation. In addition, the publicly available GEO and TCGA datasets were used to confirm that finding. The expression of *KCNMA1*can be reactivated by pharmacological demethylation, which inferred that promoter methylation is the primary mechanism for the silencing of *KCNMA1* in GC.

The clinical outcome of GC generally depends on the aggressiveness of individual tumors and growth status. TNM stage is still the critical clinical factor that influences the prognosis of cancer patient. However, recurrence of many GC patients often occurs at early stages. Identifying additional prognostic makers, which can provide better risk assessment to extend survival, is necessary and crucial. We explored the clinical importance of *KCNMA1* methylation in 91 patients with GC, and found *KCNMA1*methylation was an independent predictive biomarker of unfavorable outcome in patients with GC by multivariate Cox regression analysis. Many studies have indicated the promoter methylation can serve as a promising prognostic biomarker in gastric cancers [5, 15–17]. Our findings show that *KCNMA1* hypermethylation may act as a new valuable marker for predicting the prognosis of patients with GC. *KCNMA1* was uncovered to be commonly downregulated in patients with GC, which implied the key role of the functional silence of *KCNMA1* because of promoter methylation during carcinogenesis. In this study, we have not found the difference of methylated *KCNMA1* between intestinal and diffuse tumor types, which meant the *KCNMA1* may be not involved in the lauren classification.

We further investigated the putative tumor suppressor function of *KCNMA1* in human gastric cancer both in vitro and in vivo assays. Compared with empty vector transfection, ectopic expression of *KCNMA1* in the down-regulated MGC803 and BGC823 cells significantly suppressed cell viability and reduced colony formation ability. Moreover, MGC803 and BGC823 cells of over-expressing *KCNMA1* showed significantly decreased ability in invasion and migration and suppressed the growth of subcutaneous xenograft tumors in nude mice. The mechanism by which *KCNMA1* suppressed malignant behaviors of the gastric cancer cell was mediated by inducing cell apoptosis. The apoptosis by *KCNMA1* was associated with the focal adhesion kinase (FAK), also named PTK2, which is a cytoplasmic protein tyrosine kinase. *PTK2* can enhance tumor progression and metastasis through effects on cancer cells, as well as stromal cells of the tumor microenvironment [18–20]. The kinase-dependent and kinase-independent functions of *PTK2* moderate cell movement, invasion, survival and cancer stem cell self-renewal [21]. We found the *KCNMA1* down-regulated the expression of *PTK2*, and promoted the apoptosis of GC cell lines.

The role of PTK2 as a major player in suppressing the apoptosis of cancer cell has been well revealed, and *PTK2* is often expressed at aberrant high levels in cancer cells [22–24]. Studies have identified its downstream target PI3K-AKT pathway was involved in the functions of various kinds of cells including apoptosis [13, 25, 26]. Moreover, emerging studies have confirmed the interaction between the KCNMA1 and PI3K [27]. Our research revealed the molecular mechanism that the *KCNMA1* can moderate the *PTK2*. This present study showed the significantly reduced cell proliferation, invasion and metastasis by *KCNMA1* were related to induction of apoptosis, in which the *PTK2* play a crucial role. We proposed that aberrant *KCNMA1* expression can disturb the K^+^ channel function, and thus activate the FAK pathway, which play a key role on the cell apoptosis [21]. However, it needed further functional studies to identify.

Some studies have explored the mechanism of *KCNMA1* in the tumorigenesis. KCNMA1 protein (also named BK) was the pore-forming α subunit of the α-subunit of the large conductance, voltage and Ca2^+^-activated K^+^ channel, and was thought to play several roles in cancer biology [28–31]. BK channels can promote growth and spreading of breast, prostate and gliomas tumor [32–35]. Some studies found that BK channels do not participate in glioma cell division [36] and genetic knock-down of BKα assist osteosarcoma development [37]. So the role of BK channel in human tumor may play a very complex one. In the above study, the researchers identified the *KCNMA1* generally acted as oncogene. However, in this study we found the *KCNMA1* was down-regulated in the tumor tissues due to the methylation of promoter and played a tumor suppressor role. This finding uncovered the possible new mechanism that *KCNMA1* was involved in carcinogenesis.

## Conclusion

In conclusion, we have identified a novel tumor suppressive gene, *KCNMA1*, which is frequently inactivated in gastric cancer because of promoter methylation. *KCNMA1* exerts a tumor suppressive function by regulating the *PTK2* expression to activate the PI3K-AKT pathway. In addition, promoter hypermethylation of *KCNMA1*may serve as a potential prognostic biomarker in patients with gastric cancer.

## Additional files

Additional file 1: Table S1. Clinical characteristics of 12 gastric cancer cases selected in microarray analysis. **Table S2.** Sequences of primers used in RT-PCR and MSP assay. (PDF 113 kb)

Additional file 2: Supplementary materials. (PDF 82 kb)

Additional file 3: Supplementary figure. (PDF 387 kb)

## Abbreviations

BSP: Bisulphite sequencing
*FAK*: Focal adhesion kinase
GC: Gastric cancer
*KCNMA1*: Potassium channel, calcium activated large conductance subfamily M alpha, member 1
MSP: Methylation-specific PCR
