# Supplementary material for: KCNMA1 cooperating with PTK2 is a novel tumor suppressor in gastric cancer and is associated with disease outcome
Source: Mol Cancer. 2017 Feb 23;16:46. doi: 10.1186/s12943-017-0613-z (PMC5324255; doi:10.1186/s12943-017-0613-z)
Supplement: Supplementary file 1 — Clinical characteristics of 12 gastric cancer cases selected in microarray analysis. Table S2. Sequences of primers used in RT-PCR and MSP assay. (PDF 113 kb) [file 12943_2017_613_MOESM1_ESM.pdf]

Supplementary Table 1: Clinical characteristics of 12 gastric cancer cases selected in microarray analysis

ID	Age	Gender	TNM
1	77	Male	IIIB
2	67	Male	IIA
3	37	Male	IIIB
4	64	Male	IIB
5	63	Female	IIIA
6	36	Male	IV
7	71	Male	IB
8	67	Female	IIIB
9	68	Male	IV
10	42	Male	IIB
11	36	Male	IIIA
12	69	Female	IV

Supplementary Table 2: Sequences of primers used in RT-PCR and MSP assay

	Sense (5'-3')	Antisense (5'-3')
KCNMA1-RT-PCR	TGGCCTCCTCCATGGTGA	TTCTGGGCCTCCTTCGTCT
PTK2-RT-PCR	GCTTACCTTGACCCCAACTTG	ACGTTCCATACCAGTACCCAG
GAPDH-RT-PCR	AAGGTGAAGGTCGGAGTCAAC	GGGGTCATTGATGGCAACAATA
KCNMA1-MSP-M	GCGTAGTATTTTGGAGAGAGGC	GCTAACTCGCCTAAACTCCG
KCNMA1-MSP-U	GGGTGTAGTATTTTGGAGAGAGGT	ACTAACTCACCTAAACTCCAAA
KCNMA1-BSP	TTAATAATGGATAATYGGTTTTT	CRCCCRCTCCAATTACTA
siPTK2-2	GGUCGAAUGAUAAGGUGUA dTdT	GGUCGAAUGAUAAGGUGUA dTdT
