# Supplementary material for: KCNMA1 cooperating with PTK2 is a novel tumor suppressor in gastric cancer and is associated with disease outcome
Source: Mol Cancer. 2017 Feb 23;16:46. doi: 10.1186/s12943-017-0613-z (PMC5324255; doi:10.1186/s12943-017-0613-z)
Supplement: Supplementary file 2 — Supplementary materials. (PDF 82 kb) [file 12943_2017_613_MOESM2_ESM.pdf]

Supplementary Material

Western blot analysis

After transfected with KCNMA1 or NC vectors, total cells were lysed in RIPA buffer mixed with 0.5% PMSF. They were then incubated on ice and blended completely for 30 min. After centrifugation at 12,000 g for 15 min, the supernatants were collected as protein samples. The protein samples were separated in 10 % SDS–PAGE gels, transferred onto PVDF membranes (Millipore, Billerica, MA, USA), and then blocked with 5 % non-fat milk for 2 h. Then the PVDF membranes were incubated with rabbit monoclonal antibody to KCNMA1 (Abcam, Hongkong) at 4 ℃ overnight, and with anti-mouse secondary antibody at room temperature for 2 h. After washed with TBST for 2 h, the membranes were visualized with enhanced chemiluminescence. β-actin was used as an endogenous protein for normalization.

Cell proliferation assay

Approximately 5.0×10^3 MGC-803 and BGC-823 cells were plated in 96-well plates after transfected with KCNMA1 overexpression or NC vectors. Twenty-four hours, 48h and 72h later, cell proliferation was measured using the Cell Counting Kit-8 (Beyotime, China) according to the manufacturer's protocol. The absorbance was assessed at 450 nm using the Infinite M200 spectrophotometer (Tecan, Switzerland). All experiments were performed in triplicate. The curves of cell proliferation were plotted using the absorbance at each time point.

Cell migration assay

To detect the effect of KCNMA1 on MGC-803 and BGC-823 cell migration, we performed transwell assay. Twenty-four hours and 48 h after transfection, cells were

harvested and 1 × 10^5 cells were seeded into the upper chamber of the trans-well (pore size, 8 μm; Millpore) with 100 μl of serum-free medium. Medium containing 10% FBS were added to the lower chamber. After 12 h of incubation at 37˚C, cells remaining on the upper membrane were removed by wiping with a cotton swab. Cells that migrated through the membrane were fixed in 95% methanol for 20 min, stained in 0.1% crystal violet for 15 min and then the membrane was subjected to microscopic inspection (original magnification, × 200). Cell migration was measured by counting cells in five fields per membrane and experiments were independently repeated three times.

Cell invasion assay

Similar to migration assay, invasion assay was also done in transwell chamber. One day before cells seeded on the upper chamber, all the trans-wells were spread by Matrigel (Becton Dickinson Labware, Bedford, MA). The following process of cells seeding was the same as that in migration assay. After incubation, cells remained as well as the Matrigel coating on the upper surface of the filter was wiped off. Finally, invaded cells were observed and counted following the steps described in migration assay.

Annexin V apoptosis assay

Apoptosis was assessed by flow cytometry after staining with Annexin V (FITC-conjugated) (BD Biosciences, Erembodegem, Belgium) and 7-amino-actinomycin (7-AAD; BD Biosciences).
