# Supplementary material for: KCNMA1 cooperating with PTK2 is a novel tumor suppressor in gastric cancer and is associated with disease outcome
Source: Mol Cancer. 2017 Feb 23;16:46. doi: 10.1186/s12943-017-0613-z (PMC5324255; doi:10.1186/s12943-017-0613-z)

Supplementary Figure legends

Supplementary Figure 1 The difference of KCNMA1 methylation and expression between the gastric carcinoma and paired corresponding normal tissues in The Cancer Genome Atlas (TCGA) and Gene Expression Omnibus (GEO) databases. **(A)** The beta-value of cg24113782 in gastric carcinoma and paired corresponding normal tissues in TCGA. **(B)** The beta-value of cg04688368 in gastric carcinoma and paired corresponding normal tissues in TCGA. **(C)** The expression of KCNMA1 in gastric carcinoma and paired corresponding normal tissues in TCGA. **(D)** The expression of KCNMA1 in gastric carcinoma and paired corresponding normal tissues in GEO (GSE2685). N, normal tissues, T, gastric carcinoma.

Supplementary Figure 2 Methylation and mRNA expression of KCNMA1 in the GC cell lines after 5-Aza-dC. **(A)** The mRNA expression of KCNMA1 in 4 GC cell lines (MKN28, BGC823, SGC7901, MGC803) and gastric mucosa cells (GES1). **(B)** and **(C)** After treatment with 5-Aza-dc, the mRNA expression of KCNMA1 is changed，B and C shows MGC803 and BGC823, respectively.

Supplementary Figure 3. Interaction network for KCNMA1 and PTK2. A, The black nodes stand for KCNMA1 and PTK2, respectively. The red nodes stand for genes in TCGA data, which may interact with KCNMA1 or PTK2. B, The expression correlation of KCNMA1 and PTK2 was verified in TCGA (n= 289).

Supplementary Figure 4 Interfering efficiency of three si-PTK2 on knockdown of coding gene PTK2. Among them, si-PTK2-2 has the highest inhibition ratio.

Supplementary Figure 5 *In vitro* the tumorigenesis effect of PTK2. (**A)** si-PTK2 significantly inhibited cell viability in MGC803 cell. **(B)** si-PTK2 remarkably suppressed migration ability of MGC803 cell.

Supplementary Figure 1

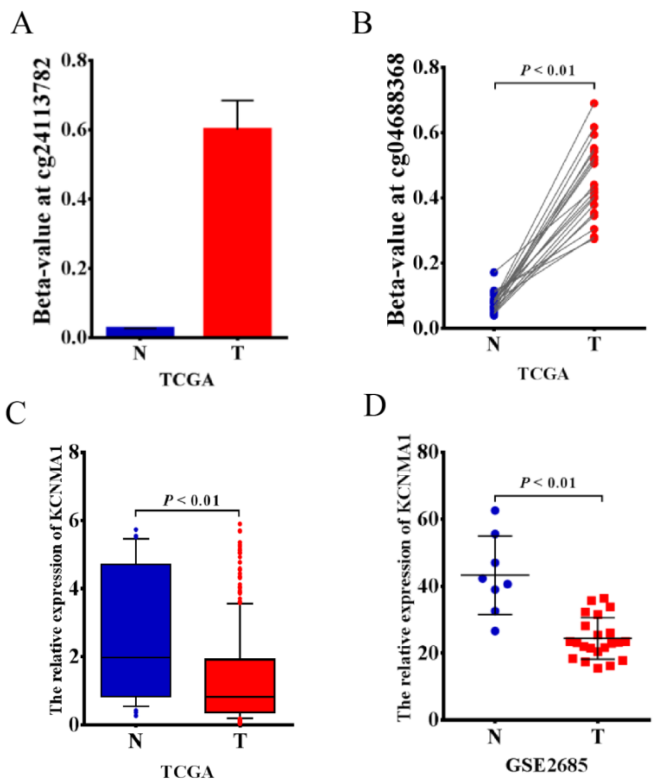

Supplementary Figure 2

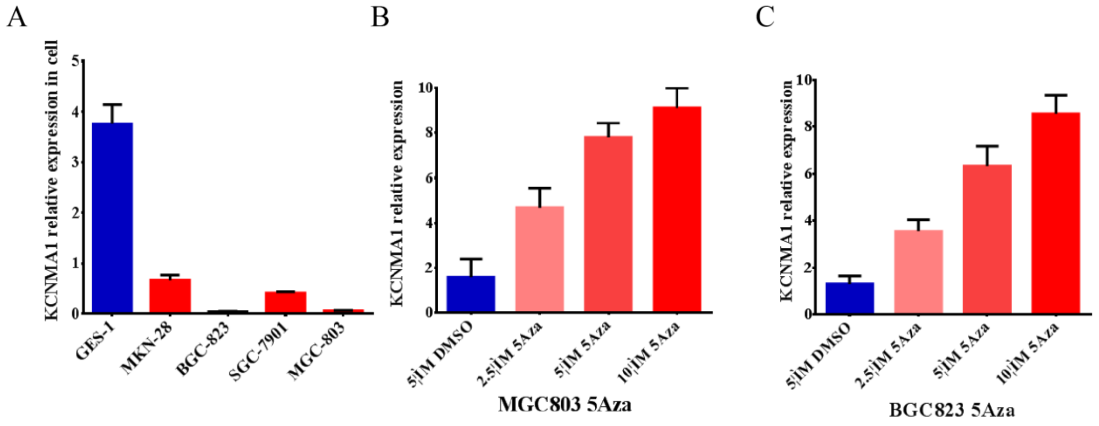

Supplementary Figure 3

A

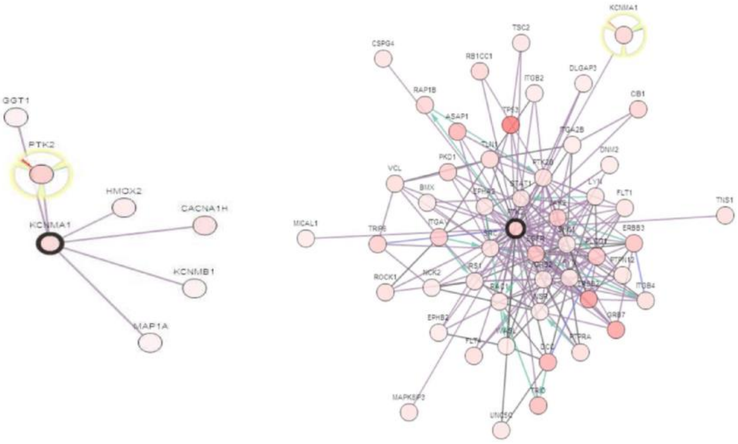

B

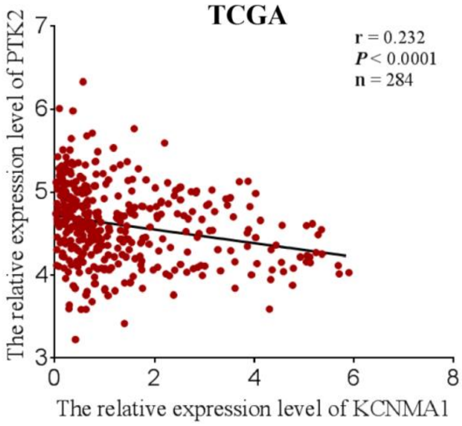

Supplementary Figure 4

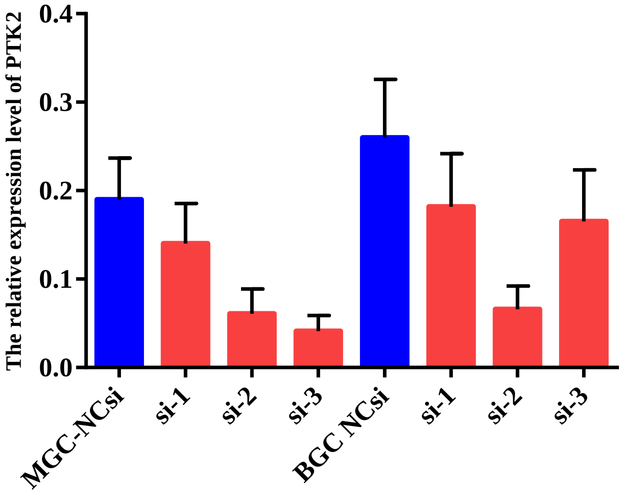

Supplementary Figure 5

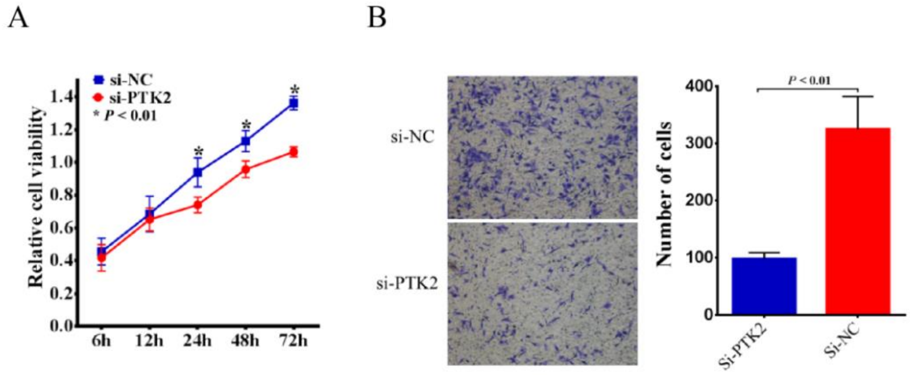

Supplement: Supplementary file 3 — Supplementary figure. (PDF 387 kb) [file 12943_2017_613_MOESM3_ESM.pdf]
